# Impact of discrimination experiences on cognitive test performance in a multiethnic cohort

**DOI:** 10.1002/alz70857_105921

**Published:** 2025-12-25

**Authors:** Emily Post, Genna M Mashinchi, Taylor F Levine, Jessica ZK Caldwell

**Affiliations:** ^1^ Cleveland Clinic Lou Ruvo Center for Brain Health, Las Vegas, NV, USA; ^2^ Cleveland Clinic, Las Vegas, NV, USA; ^3^ Nevada Exploratory Alzheimer's Disease Research Center, Las Vegas, NV, USA

## Abstract

**Background:**

Greater psychosocial stress relates to poorer cognition, especially executive functioning (McManus, et al. 2021). Previous studies have found that racial discrimination impairs cognitive performances (Barnes et al. 2012); however, other forms of discrimination remain underexplored. The present study assessed how experiences of discrimination—including gender, age, ancestry, religion, sexual orientation, and physical appearance‐based discrimination—relate to cognitive performance in a large sample of Black American (BA), Hispanic/Latino (HL) and non‐Hispanic white (NHW) adults.

**Method:**

Data were obtained from 1647 participants from the Health and Aging Brain Study‐Health Disparities (HABS‐HD; mean age=63.72). The sample included three cohorts (909 BA, 406 HL, and 359 NHW) who completed a baseline visit with cognitive testing. Spearman correlations as well as general linear models (GLMs) controlling for age, sex, and education assessed the relationship between self‐reported discrimination experiences (Everyday Discrimination Scale [EDS]), cognitive test scores (Spanish English Verbal Learning Test [SEVLT] learning, SEVLT delay, and Trail Making Test Part B [TMTB]), and functional independence (Clinical Dementia Rating‐ total score [CDR]). We hypothesized that greater discrimination experiences would predict poorer cognitive and functional performance.

**Result:**

Counter to expectations, in the BA cohort, analyses showed weak but significant correlation between greater EDS and better scores on TMTB (r_s_=‐.151, *p* = .004) and SEVLT delay (r_s_ = .104, *p* = .048). In the NHW cohort, higher EDS related very weakly but significantly to better scores on SEVLT learning (r_s_ = .094, *p* = .005) and TMTB (r_s_=‐.096, *p* = .004). In the HL cohort, a weak yet significant correlation was observed between higher EDS and worse CDR outcome (r_s_ = .140, *p* = .005). GLMs showed higher EDS predicted worse CDR score in HL individuals after controlling for age, sex, and education, but did not predict scores in other groups.

**Conclusion:**

The present findings suggest discrimination may have specific impact on functional decline in HL individuals. Contrary relationships in BA and NHW cohorts are of unclear significance. Overall, results support the idea that discrimination impacts cognition across different racial and ethnic groups in complex ways and study in a broader context with additional social determinants of health and resilience factors is warranted.

